# On the Death Wound of General Abercrombie

**Published:** 1804-11-01

**Authors:** 


### *To the Editors of the Medical and Physical Journal.*

Gentlemen,

THE importance of the present subject, as well as its dignity, impresses an anxious solicitude in the breast of every British Soldier, and exacts a due reverence to the object of universal adoration. No doubt can be entertained, that the statement of the particulars of the mortal wound which caused the death of the gallant Abercrombie would be received throughout the medical department of the army with every demonstration of an additional advantage to science, while the community at large would treasure every document as the invaluable memento of an unparalleled display of valour and philosophic resignation when in the bosom of victory, undaunted at the approach of impending dissolution.

 The

The observations of the *real* (vox et prætcrea nihil) Campaigner have neither the shape of an answer to those of the old Campaigner, nor have they afforded the smallest elucidation to the subject so expressly inquired for.

In my perusal of the old Campaigner's observations, I find nothing like the expression of "wonder that the old General should have fallen a victim, &c." and I think, that if it was possible for the old Campaigner to wonder at all at the dispensations of Providence, the *wonder* would arise from his conviction of the wide disparity in the functions and abilities of *two* individuals, wherein the most worthy and efficient was allotted as the victim.

The *real* Campaigner has very unguardedly thrown an imputation, not of the happiest kind, upon the high professional character of the Field-Inspector, as also upon Mr. Gilham himself. He states, that the Field Inspector did not arrive until after Mr. Gilham had commenced the operation for extracting the ball, and that he then differed in opinion from Mr. Gilham; in consequence, "the General was taken to the Fleet, when he died, &c." It is asserted, that Mr. Gilham had so far proceeded in the operation as to feel the ball very distinctly; if so, why was the operation for extracting it deferred? or, on such an occasion, does it not appear even criminal to delay in the least such a necessary proceeding? What motive could induce any difference in opinion when the ball was distinctly felt by the tactus eruditus of Mr. Gilham; or, what obstacle presented itself, which impeded the progress of the operation, remains yet for further explanation.

I wish further to observe, that as Mr. Gilham so distinctly felt the ball, the incision must have been very extensive; and as the vessels of the limb are very great and numerous, and their situation or vicinity to the neck of the thigh-bone, in which it is said the ball was found after death, requiring much circumspection and dexterity in the operation, it is certainly of great importance to know, what vessels were divided by incision, what parts were wounded, lacerated, or contused, by the entrance of the ball, the quantity of blood lost and effused, and many other important particulars, which I trust will be given to the public. If Mr. Gilham supposes that the medical department of the Army, and more particularly the Staff on the expedition, are so disinterested concerning the history of the case alluded to, as he imagines the public to be; I beg leave to inform him, through the medium of your your Journal, that a contrary disposition exists throughout, and that the particulars, as well as the removal of the imputation upon professional characters, will meet the approbation of,

Yours, &c.

PHILOMILES.

*St. James's Street, Sept.* 12, 1804.

